# Anæsthesia and Anæsthetics

**Published:** 1879-08

**Authors:** J. J. Caldwell

**Affiliations:** Baltimore, Md.


// THE DENTAL REGISTER.

VOL. XXXIII.] AUGUST, 1879. No 8.

### ANÆSTHESIA AND ANÆSTHETICS.

BY DR. J. J. CALDWELL, OF BALTIMORE, M. D.

*Read before the Dental Association of the State of Maryland and the District of Columbia.*

[Continued from last number.]

"From my own experience and observation, I can readily see how there can be seven causes for death during the administration of anæsthesia, even when the chemically pure drugs are used, the patient being in a recumbent posture, with loose clothing and in a proper position for its inhalation. Etherization in the sitting posture is dangerous and should never be undertaken. May not the perfect safety with which chloroform is used in obstetrical practice depend much upon the empty condition of the stomach, the loose clothing and the horizontal decubitus, which are considered the best preparation for safe inhalation, accompanied, as it always is in labor cases, with no fear of, but a longing for, the anæsthetic.

"In the first place, I can understand how a cloth saturated with chloroform, covering the face of the patient so as to exclude air, may necessitate the inhalation of so saturated an atmosphere as to cause excessive irritation of the air passages. This may act injuriously upon the respiration and circulation. Should a fatal result occur under this condition of faulty administration, we would not call this a death from chloroform under careful use, but its careless abuse; a result that could not occur if common precautions are taken—such precautions as every physician is expected to use who administers any of the efficient drugs of the materia medica. Should a physician intending to administer hypodermically a few minims of Magendie's solution from a full syringe carelessly inject the entire contents under the skin, no one would attribute the blame to the morphia, but to the hand that worked the syringe. In this case life is sacrificed by carelessness in the use of a remedy known to be potent, and therefore one to be used with caution.

"A second cause of death may readily occur with the early administration of both ether and chloroform. In many cases, the local action of the inhaled vapor so irritates the throat as to cause contraction in the tongue muscles and a closure of the laryngeal opening by the close approach of arytenoid cartilages and the overlapping of the epiglottis. This condition is evinced by stertorous breathing, change of countenance, discoloration of face, impeded thoracic movements, and vain attempts at thoracic expansion. The patient is threatened with asphyxia by an involuntary but successful effort at partially swallowing his tongue. Should he be allowed to remain in this condition, death will as surely take place as when one is seized by the throat and fatally strangled. If the mouth be opened, tongue caught by the tongue-forceps and drawn forcibly outward, to one side, so as to elevate and advance the epiglottis and separate the arytenoid cartilages, thereby re-establishing the laryngeal opening, a loud, sonorous inspiration is immediately heard, air pours into the lungs and the ominous cloud upon the face at once passes away. Should there be no tongue-forceps at hand, and the drawing out of the tongue be omitted, we would have, under these c[illegible]umsta[illegible]es, another glaring instance of death from maladministration of the anæsthetic. A surgeon who is not prepared to protect his patient from this accident, while inhaling an anæsthetic, would have as good reason for escaping censure as he who, after an amputation, found that he had omitted to procure the instruments necessary for securing the arteries, and, hence, allowed the patient to die from hemorrhage.

"A third cause of danger in the inhalation of anæsthetics resides in the inexperience of the administrator or in his inattention. I have already said that the physician who administers an anæsthetic has full occupation in the part assigned to him, and should not permit his attention to be diverted to the surgical work performed by another, and for which he is preparing the patient. I have seen instances in which the administrator of the anæsthetics, often a student of medicine, in his eagerness to follow the several steps of the operation had become so oblivious to his responsible trust as to allow the thickly-folded cloth to compress firmly both nose and mouth of the patient, who, with asphyxia imminent, was too insensible and powerless to push away the obstacle and escape the threatened suffocation. Had the patient a feather pillow pressed over the face he could not be more surely suffocated than by this thickly-folded towel. Many a time in doing surgical work have I pulled the cloth from the face to the immediate relief of the respiration. In ophthalmic surgery, to which my work is now exclusively confined, this accident can not occur as the face is constantly under my observation. Had the careless administrator been permitted to smother his patient we would have had a death announced from chloroform, while a towel which had never known chloroform if applied in a similar way would have done equally efficient work. Another clear case of careless administration.

"Still a fourth cause will be wrongfully attributed to the anæsthetic, should it occur during the act of vomiting. If the patient be allowed to remain upon his back during emesis, and be not rolled over upon his side or face, some of the contents of the stomach will pass downwards from the pharynx into the trachea and cause death by suffocation. Autopsies are not often thoroughly made after supposed death by anæsthetics. Usually the brain, the heart, and the kidneys are examined, and, possibly, the stomach, liver, and the bladder, but not the throat and the larynx. But in one recently reported death during chloroform-inhalation, in which a patient suddenly expired immediately after the act of vomiting, the larynx and the trachea were found packed with food ejected from the stomach. In another recent case at the University College Hospital, London, of death following vomiting under ether during an operation for strangulated hernia, the autopsy exhibited stercoraceous matter in the trachea and right bronchus. Again, a case of death at the hands of a careless administrator in his bad manipulation of the patient, and not from the anæsthetic.

"The fifth cause of death from both chloroform and ether, probably the most common of all, has been more active of late years, being rendered so by the unsettling of the former confidence which surgeons have had in the safety of anæsthetics. In the present timidity, surgeons do not now push the inhalation to the degree of suspending the functions of such parts of the cerebro-spinal system as preside over the emotional, sensational, motor and reflex acts; the only condition recognized as one of perfect safety in chloroform and ether anæsthesia. I refer to that condition in which peripheral irritation can no longer be transmitted through the cord to the brain, and then back, by the vagus and peumogastric nerves, to the cardiac ganglia. Any condition short of this stage of temporary suspension of reflex agencies leaves the heart exposed to those serious inroads from peripheral irritation through which its movements may be suddenly and permanently arrested. Such fatal results are identical with instances of nervous shock, so familiar to operators in former times, and then deemed a sufficient explanation of death under the circumstances. In this way can be satisfactorily classified the many deaths under anæsthetics for trivial operations, as tooth-drawing, opening of abscesses, etc., when only enough of the agent was inhaled in the sitting posture to partially stupefy, but not to protect against reflex accidents from emotional or peripheral excitement.

"In this class are placed those many fatal cases during the more serious operations, in which the timidity or anxiety of the surgeon, with unsettled confidence in the article he is using, induces him to arrest the inhalation before the period of safety has been reached. He commences the cutting operation with the vital organs all exposed to injurious reflex peripheral irritation, and under the cloak of an anæsthetic, without its protection, invites disaster. Hence it is that an operator who has once been frightened by anæsthetics, continues to have accidents which do not occur to others who, never having seen trouble, administer the drug boldly. Timidity here must be classified with ignorance, both being dangerous negative qualifications for successful surgery. When deaths occur under these circumstances, the fatal result is not to be attributed to the anæsthetic, but, on the contrary, to the want of it; clearly a defective administration, induced by unwarrantable and unworthy fright on the part of the operator.

"Under this heading I would also class the deaths said to be occasioned by heart diseases under the inhalation of chloroform. Richardson, of London, after a critical examination of the various diseased conditions of the body said to be hostile to the administration of chloroform, and, especially, after maturely considering the many varieties of heart disease, including valvular growths, vascular contractions, cardiac, hypertrophies, stenosis of heart orfices, and, in fact, the entire list of heart troubles, sums up his experimental observations with the following remarks: 'On the whole, the only diseased condition which I could give as a warning to practitioners from exceptional danger in the administration of chloroform is the diagnostic of a dilated and a weak left heart.' And then states that in one such case he had forewarned the surgeon, who then gave chloroform and had a fatal result. But what guarantee have we that the patient with such a heart would not have succumbed to the operation itself? These heart conditions are so prone to syncope that anæsthetics are needed during painful operations to prevent the fatal emotional shock. In such cases we recognize the necessity for cardiac stimulus, whisky, which may have prevented the fatal issue.

"Diseased conditions of the heart, regardless of kind, may make this important organ peculiarly susceptible to syncopetic influences when reflex action has full sway; hence, we find violent emotional excitement a fruitful cause for mortality in the subject of heart disease. Many such persons having to undergo painful surgical operations in former times, before the introduction of chloroform, suddenly collapsed with the first incision; and they still die as of old when they are not properly protected by complete anæsthesia. Should chloroform be freely given to patients with heart disease, regardless of kind, who must submit to painful operations for the cure of some surgical affection, by its liberal use they are put in a condition of safety against all emotional and reflex annoyances, without which they could not escape trouble.

"I look upon chloroform as the strong bridge which will conduct patients suffering from serious heart disease safely through serious operations. As a surgeon in large ophthalmic practice, I frequently am compelled to perform the most delicate and painful operations upon the eyes of timid patients suffering from heart disease in its various forms. Cataracts occurring usually at an advanced age, most frequently between sixty and eighty years of age, are often associated with organic diseases of the heart in patients enfeebled by sensibility. I never refuse to give such patients chloroform; on the contrary, I urge its use. The only difference that I make in such cases over other patients is, if possible, by exercising more care in establishing the safe stage of complete anæsthesia through the liberal use of the drug. From the standpoint of my own personal experience, I know of no organic lesion which contraindicates the careful and thorough administration of chloroform.

"The sixth cause of death during the administration. No one can doubt for a moment that chloroform and ether possess toxic action, and that, in common with all other active agents in medicine, the danger is dependent upon the size of the dose used; also, that the dose of an anæsthetic can be made large enough to kill, by enfeebling and, finally, paralyzing the nerve centers from which the heart and lungs draw their inspiration. This class of remedies is clearly cumulative, and, when enough has been inhaled to cause the suspension of voluntary motion, sensation, and reflex action, if their adminstration be continued, instead of being suspended, an amount can be concentrated in the circulation quite sufficient to stop respiration and the heart's action.

"As I have said before, there is a broad gulf between that degree of anæsthesia which only suspends so much of cerebral action as still allows full play to the vital organs—a perfectly safe condition for surgical operations—and that fatal overdose of the anæsthetic from which there is a suppression of the cardiac centers of innervation. Surgeons often employ ignorant assistants to administer the anæsthetic, who conceive it to be their duty to keep the saturated cloth to the nose of the patient, and they know no stopping point. They are told to give the ether, and they show neither judgment nor discretion. Unless watched by the surgeon, whose attention should be concentrated elsewhere, they go on applying the vapor in spite of complete narcotism. Fortunately, the elimination of the gas from the blood through the lungs is so rapid that, by removing the towel a very few minutes, expirations will reduce the amount in the system to a safe standard and dissipate the threatened dangers. When, through this overdose, the vital functions are suspended, the killing should be put to its proper cause—an overdose through ignorance; another clear case of maladministration.

"'The effects of anæsthetics,' says Claude Bernard, 'depend on the immediate influences exerted by the drug upon the sensitive elements of the nerve centers, in virtue of which their properties are temporarily suspended. In cases of idiosyncrasy, these functions, so essential to life, become permanently suppressed, and yet, with the death of the individual, the changes in the nerve elements are of such a nature that so far they have altogether escaped detection by pathological investigators."

Says Chisholm again:

"I will add my own individual experience. I have been practicing surgery twenty-five years, and have used chloroform largely during that entire period in private and hospital practice, in the army as well as in civil life, and have administered it to the extent of fully six thousand cases. For some years I have administered it on an average of at least once every day. I have given it to the very young and to the very old; to the very strong as well as to the very weak; to the healthy as well as to the extremely diseased, regardless of the organ in which the trouble may be located. I have seen patients thoroughly anæsthetized by a half-drachm of chloroform, and I have used an entire pound before I could secure full narcotism, and I have had occasion to keep up the anæsthesia as long as three hours at a time. I have accepted Syme's axiom, and given chloroform to every one, regardless of visceral complications, who has applied to me for a serious surgical operation, and I have yet to see the first death, either in my own practice or that of my friends.

"Now let us sum up the evidence which I have collected, and here we find an array of over two hundred and fifty thousand administrations of chloroform with twelve deaths; even attributing them all to idiosyncrasy, which calls for a most unbounded charity, and we only have one death in twenty thousand cases. Can any stronger proof of the excessive rarity of the fatal idiosyncrasy in chloroform be needed?

"There is no remedy really good in medicine which is not capable of mischief. More persons are killed every year from the abuse of opium than have been attributed to chloroform from its discovery even to the present time. Deaths from opium in England alone number three hundred a year. How many hundreds are to be added to this, occurring under the careful administration of skilled physicians, we can easily conjecture. Yet who proposes to substitute for this king of drugs any milder narcotics? We are ready to accept the comparatively small fatality for the immense good it accomplishes. How often, during the summer, do we find in the daily papers reports of healthy persons dying suddenly from the too liberal potation of ice water, yet who would listen with any patience to the so-called philanthropist who would wage war against this universal beverage? It has been stated that more persons are killed by slipping on fragments of orange peel in the streets of London than from the inhalation of chloroform, No one suggests the destruction of orange groves on this account; no one finds fault with the laws of gravitation because persons continue to fall down and break their legs or neck. It is unfortunate for the individual, but the great laws of nature must ever go on, and the great designs of the Creator enjoyed with wonder and astonishment. Shall we give up the use of horses or steam because accidents happen from both? Would we have all the rivers dried up because now and then a drowned man is fished up out of them? The great discoveries are for the good of the many, not for the few? You can not avoid every danger and supply every good.

With anæsthetics, the little risk, infinitesimally small, is immensely counterbalanced by the protection from the very many dangers during operations, so well known and so often experienced when operations were performed without them. Chloroform, when judiciously used, is one of the safest active remedies of the materia medica, supplying nearly every good and avoiding nearly every danger.

"The greatest gift of God to man through natural science is chloroform."

Says Hodgkin:

A resume of the attitude of the professions of medicine and dentistry on the subject of anæsthetics and their value and action may here be given and as dentists you are naturally so strongly interested in the subject, as to justify an expectation that this will be complete, and yet it is a difficult thing to do; for science, though so exact, is yet variant in the enunciation of her dogmas through her chosen apostles.

The unanimity of division between the northern and southern sections of our land on the relative dangers of ether and chloroform is well known. The wave which seemingly threatened the destruction of the chloroformites was driven back by the accident of the late Civil War, which proves that the large percentage of deaths at the hands of those using chloroform in civil hospital cases, did not occur in the field and under average war circumstances. The advocates of ether still cling to that drug as THE anæsthetic, claiming for it exemption from fatal effects in all but foregone cases.

From the pen of Prof. J. J. Chisholm, of Baltimore, and Dr. Sigismund Waterman, of New York, have lately appeared original and thoughtful articles with information from recondite sources, on the subject of chloroform by the first named, and on the action of anæsthetics on the blood, by the latter, which are well worthy of a careful study by the surgeon. Most of you are so familiar with both of these papers as to justify on my part the assumption of complete possession of the salient points of these exceedingly interesting productions; yet that I may refresh your memories, I recall their main features.

In all cases of fatal or untoward result from the administration of anæsthetics, and in the investigation of the causes which lead to casualty, we must carefully weigh the probable difference in handling cases in ordinary hospital and city practice, and surgical cases occurring in field practice.

Is the proper study of the field of anæsthesia found in the previous condition of the patient? Is it surgical shock which kills the subject, or is it the drug? Is some obscure change in the blood itself the cause of the anæsthesia and the accidental catastrophe? Or is it that to mere incidents as to position, dress, or the method of administration, we may ascribe the untoward result?

These are grave questions to be flippantly handled or hastily decided, and all honor to those who, in a true scientific spirit, undertake their cautious, patient, slow solution.

Dr. Waterman brings to the aid of the pathologist one of the newest in application of scientific instruments in horological art—the spectroscope; and, with all the ardor of an enthusiast, attempts the demonstration of the problems of the life-in-death state of anæsthesia, as seen under nitrous-oxide, chloroform and ether. Special attention and prominence is given by him to the first named gas, the administration of which, his demonstration seems to prove fraught with danger, and which, he asserts, his clinical practice corroborates fully.

To summarize: Blood seen under the spectroscope gives certain characteristic lines as beheld in the spectrum. These, as all other spectral lines, are constant and definite. No other line could be mistaken for blood; nor could this be mistaken for any other organic substance. Oxydized blood displays a different spectrum from de-oxydized blood, and putrid blood shows a spectrum peculiar to itself, unmistakably marked. The de-oxydized blood can be restored to a normal state of re-oxydation, and the changes that play through it are sharply defined and well understood.

On the other hand, blood which has been subject to the action of nitrous-oxide gas and blood which has become putrid show precisely similar lines in the spectrum, and no manipulation can develop in blood acted upon by this gas, other lines than this "death-line of putridity, or its semblance, spectroscopically considered; and the conclusion which is reached seems an inevitable one, viz.: that the changes wrought in blood by the protoxide of nitrogen and the destructive changes which putridity sets up are identical. If there is fault with this theory, it must be set down to imperfect understanding of conditions; for the advocates and disciples of spectroscopy contend for its infallibility, and, doubtless, with justice.

We must guard this point by the observation that, while according to the spectroscope all that its most zealous advocates claim for it, we must bear in mind that its manipulators and interpreters are human, with human frailties; the most fatal of which to thorough research or candid scientific examination is the tendency to "jump to conclusions." It was Agassiz who claimed that not one man in thirty was competent to make the simplest observation; and we know the fatality which besets the most judicial of us, and from which not even a Tyndall can free himself, of being able to see only that we are looking for.

The rationale of the action of nitrous-oxide is certainly obscure. The hurried respiration, the cyanosis, the staring eyes are fearfully suggestive of partial asphyxia, and mark unmistakably imperfect decarbonization of the vital fluid. On the other hand, the rapid recovery, the occasional cheerfulness, the absence of loss of tone, and, in a word, the speedy return to normal conditions as a rule, seems to indicate that no great morbid change can have taken place by virtue of this gas, and that injury is not often apparent. The effect of these studies, however, is seen in the undoubtedly-lessening use of this gas, and, curiously, coincidently with these investigations is seen a lessening demand for it on the part of the public.

The studies of Dr. Waterman on chloroform and ether, spectroscopically, are of less moment as to satisfactory results. He considers as one of the dangers of chloroform the retention of the vapor in the lungs, poisoning, much as does carbonic acid gas, by displacement—its specific gravity being so great—and suggests as a remedy the inhalation of hot air.

The paper of Professor Chisholm, on "What anæsthetics shall we use?" is a special plea for chloroform, in which he takes the bold ground that the dangers to be apprehended from the administration of that drug are in inverse proportion to the profoundness of the anæsthetic condition, and that reflex action is the cause of danger and death. The advanced ground is taken that as surgical shock in anti-anæsthetic days was the occasional cause of death, so now the same cause inheres, and as it was then manifestly due to the reflex disturbance then, that now the same reflex action and danger are to be and can be averted by profoundness of the anæsthetic condition. That weak lungs and heart are not contra-indication of chloroform, but that they even suggest a more vigorous use of the drug; as full anæsthesia will tend to overwhelm the center of reflex action in the brain, etc., prevents disaster. This is suggestive of the commendation of Mr. Sam Weller of hot punch for warding off anticipated rheumatism; which punch, he sagely remarks, is the best thing in the world for the purpose, and, if it ever failed it was because the patient fell into the vulgar way of not taking enough of it.

This is bold doctrine certainly, and the conclusions, though founded on an extensive practice and copious in statistics, are such as most of us would shrink from. Yet we can hardly tell how far this shrinking from an acceptance of such doctrine is the effect of the previous instillment of a contrary doctrine, so potent is education in moulding our ideas of the proper thing, and so difficult is emancipation from precedent.

It is the bold who ever succeed; and that death at the surgeon's hands is due to timidity, arising from imperfect knowledge, rather than from the boldness of complete mastering of the subject, is more true than we perhaps care to acknowledge.

The horizontal position is insisted upon by all modern chloroformists as sine qua non to its safe administration; and abstinence, as a matter of course, is no less judicious. The pallor which has frightened so many, coming on at the advent of the administration is, as he suggests, often only the precursor of emesis, which, if occurring must be watched, as fatal accidents have arisen from the passage of ejecta into the trachea. Death from chloroform at the hands, or on the hands, of the dentist is, relatively speaking, much more common than with the general surgeon. The solution of this seems to be found in the fact that ænemia of the brain is induced by the drug, which assuredly profoundly depresses that organ, and the ænemia, inducing syncope, is again reflected on the heart, inducing paralysis of that center of life; then, with two legs of the great tripod of Bichat taken away, the catastrophe is precipitated. Hence, decubitus is insisted upon.

The preparation for anæsthesia is well known. The horizontal position, just adverted to; the empty stomach; the perfect freedom of respiration, guaranteed by the absolutely loose clothing; and, finally, the pre-administration of whisky. This last should be ever borne in mind, remembering that alcohol of itself is a true anæsthetic, and its usefulness in conjunction with chloroform is no longer a matter of theory. Yet the old observation of Paget, corroborated by every day experience, must be kept in view, that none bear operations so badly, and in no case does the experienced surgeon so shrink from grave operations as on confirmed inebriates.

Lastly, Dr. Hunter McGuire, of Richmond, Va., has announced, and his observations are confirmed by others, that quinia is of essential service in preventing or modifying surgical shock; a discovery of profound importance, if true, and one which will go far to relieve the mind of the sympathetic and ever solicitous surgeon, burdened as he must be with a sense of responsibility.

Dr. Comes, of Louisville, Ky., in a late paper on chloroform, thinks the chief danger from that agent arises from an accumulation of it in the system, which result, he judges, would likely occur when it is given tardily. And the weight of opinion seems of late to favor rapid, rather than slow, administration of this anæsthetic; a prompt overwhelming of the patient, rather than a slow and cautious bringing on of the unconscious condition. Caution is not at all incompatible with vigorous action, and the timid creeping of the fearful may, indeed, be a cause of unsuspected danger, injury arising out of the very means taken to prevent it. Dr. C. thinks our American people less easily overcome by chloroform than Europeans, except the Irish, and finds that the Germans and Negros yield readily to its influence.

We may sum up the present attitude of the advocates of anæsthesia in the famous words of the famous Syme, who said that he "had given chloroform for years without accident, attributes his success to two causes: first, he always used pure chloroform; and second, he always gave plenty of it." And he is followed of late by Professor Chisholm with the glowing tribute that "it is God's latest and best gift to man."

Says Harriman:

"No wonder, therefore, that at the first achievement of anæsthetics the hearts of philanthropists beat with joy, and dental and other surgeons were greatly rejoiced. No wonder, when, after repeated experiments, it became a settled fact that pain was entirely unknown to the subject of capital operations, that Oliver Wendell Holmes, M. D., a favorite (as all will attest) of science and the muses, declared: 'The knife is searching for disease, the pulleys are dragging back dislocated limbs, Nature, herself, is working out the primal curse which doomed the tenderest of her creatures to the sharpest trials; but the fierce extremity of suffering has been steeped in the waters of forgetfulness, and the deepest furrow in the knotted brow of agony been smoothed forever.' No marvel that the late John C. Warren, M. D., of Boston, a leading surgeon, who had long witnessed the terrible suffering of his numerous patients while enduring surgical operations, when he was assured of the power of anæsthetics exclaimed, "Who could have imagined that drawing the knife over the delicate skin of the face might produce a sensation of unmixed delight; that the turning and twisting of instruments in the most sensitive bladder might be accompanied by a beautifnl dream."

To all this, the dental surgeon of the days when patients dragged themselves with reluctant step to the chair might add, "How amazing that the profession can now perform their most difficult operations while they who are being racked and torn are entirely insensible!" But so it is. All honor to the great Creator who has given us the great blessings, anæsthetics!

Nitrous-oxide gas.—This gas is generated for anæsthetic purposes from the nitrate of ammonia, by the application of sufficient heat to volatilize it. Care should be taken, however, not to apply the heat in excess, for then will be found nitric-oxide, which gas is a poison. Too great heat would also force the gas through the wash-bottles at such a rate of speed as to prevent the complete absorption of the impurities it contains.

Great care should be exercised in the preparation of this gas. It should never be entrusted to novices. My method of manufacture requires four jars—one containing a solution of proto-sulphate of iron; another, a solution of caustic potash, the two other, water. As before observed, the nitrate of ammonia must be heated to a correct temperature, when will be obtained nitrous-oxide in purity. It is important that the gas should be allowed to remain over water from three to six hours, in order that the impurities which may remain in the gas after washing may have sufficient time in which to be absorbed. Thus the gas will be more powerful as an anæsthetic.

Nitrous-oxide may be condensed into a liquid. It was first exhibited in this form by Dr. Evans. It is usually contained in a strong metallic cylinder, to which is attached, by a tube, a rubber-sack or bag, to which the gas is admitted by turning a stop-cock, and the gas is administered as in the times when first introduced.

Anæsthesia may be produced by a variety of substances. Among these may be mentioned acetic, nitric, and sulphuric ether; protoxide of nitrogen, or laughing-gas, (nitrous-oxide) chloroform, common illuminating gas, naptha, carburetted hydrogen, benzine, amelque, and Dutch liquid. Of these, preference has been given to sulphuric ether, chloroform and nitrous-oxide gas, both as regards safety and efficiency. It is well to know which is the safest, and, at the same time, the best for the purposes of the dentist. This knowledge can only be obtained by experiments.

Experiments thus far have very largely proved adverse to the use of chloroform. To show the relative merits of these substances, I shall now proceed to detail the result of some experiments which I have made, and submit testimony.

Says Harriman again:

"In the month of February, 1870, placed a cat in a glass-receiver, and subjected it to the influence of chloroform. In one minute and three-quarters the animal became insensible, and in three minutes respiration ceased.

"On the same day administered nitrous-oxide to a cat; in seven minutes it was unable to stand, and at the expiration of ten minutes was removed from the jar. In five minutes after removal the animal could walk, and in twenty minutes its whole appearance was natural; there being nothing to indicate that it had been out of its ordinary condition.

"The following day, nitrous-oxide was administered to a cat; the animal being unconscious fourteen minutes. In six minutes after its removal consciousness returned. This experiment was witnessed by Dr. C. G. Davis, of New Bedford, Mass.

"Administered nitrous-oxide to a cat. The animal became insensible in two minutes and a half. Consciousness returned in eight minutes.

"The same day, placed a cat under the influence of nitrous-oxide. In four minutes it was rendered insensible, and one minute after being removed became conscious.

Subjected a cat to the influence of nitrous-oxide, the animal being in an insensible condition sixty-two minutes. The nitrous-oxide was renewed four times in this experiment, the carbonic acid gas being allowed to escape at each renewal. The same cat was under the influence of nitrous-oxide twenty-six minutes, without renewal, at the Boston Dental College.

"Administered benzole or benzine to a cat; insensibility was produced in three minutes and a half. After the removal of the anæsthetic, consciousness returned in ten minutes.

In a case of the administration of nitrous-oxide to a cat, death ensued at the expiration of eighteen minutes. The cat, as subsequent examination showed, was not in a healthy condition; the lungs being entirely out of the normal state. I am quite satisfied that, even at the eighteenth minute, the animal might have been resuscitated, but it being my original intention to cause the animal's death, I did not make the attempt.

"In the case of a cat where the chloroform had been recently administered in a glass-receiver, the animal died in three minutes. I immediately made an incision in the thoracic cavity, and found both lungs congested, and the air-cells nearly all closed. which shows the great danger of destroying life in the use of such a powerful anæsthetic.

Franklin R. Thomas, D. D. S., in a communication to the Dental Times, of Philadelphia, remarks: "Having practically demonstrated, with about eleven thousand persons, the influence of nitrous-oxide, and those having been administered indiscriminately as they presented themselves, and not a single instance so far as known, has any one sustained any ill effect from its inhalation, though many of them were known to have been suffering from chronic and organic diseases of different kinds.

In nearly four thousand cases where I have administered the gas, I have seen no bad effects. and I have taken pains to ascertain that it has been given more than two hundred and fifty thousand times with great success. Anæsthetics have been applied for the alleviation of pain, when necessity demanded the performance of the severest surgical operations; is an unspeakable blessing; it has gone forth to cheer and gladden humanity, but when administered, it should be done watchfully, carefully and judiciously.

The results of the electric current in the resuscitation of cases suffering from the poisonous effects of opium and other narcotics, as well as of suspended animation from, etc.

I would call the attention of practitioners to the necessity of greater care and caution in the restoration of suspended vital functions. And, in this connection, I beg leave to refer to some of the wonderful results of the application of electricity (Faradism) in the accomplishment of such restoration —to the theory of the application of the current in these cases; and to the necessity of proper batteries and instruments being placed in every dental office, in all life-saving stations, police headquarters, hospitals and other institutions; while I shall also briefly refer to the kind of battery most to be recommended.

Case I.—In the latter part of the summer of 1873, I was called late in the night to see a little child, Jennie C., suffering from a poisonous dose of laudanum. The messenger bore a note from her professional attendant, Professor Murray, of this city, stating that, as he had exhausted all other means without benefit, he had concluded to try Faradism, at the same time inviting me to join him with my battery. We found the patient to be deeply narcotized from an accidental dose of laudanum, taken some twelve hours previously.

The current was passed by placing the positive pole over the pneumograstic nerve at the angle of the sterno-cleido-mastoid muscle, whilst the negative pole was placed upon the epigastrium. A powerful primary current was applied and continued for more than three hours, with the happy result of complete restoration, encouraging and urging the respirations from eight or nine up to eighteen or twenty per minute, at which point the pupil became dilated, the pulse normal, and the sensorium capable of acknowledging the previous remedies. Hence, vomiting and purging followed, and the patient's life saved thereby. Here we present a typical cause of profound narcosis, as well as the antidote.

We were induced, in this as in many similar cases, to try the efficacy of the current from the interesting experiments made upon living rabbits, detailed by Dr. Wilson Phillips, in his dissertations in the "Philosophical transactions," at the London Academy of Science. In one instance, after incision in neck, severing the pneumograstic nerve, the parsley that had been eaten remained in the undigested state; and, after evincing much difficulty in breathing, the animal seemed to die of suffocation. In other rabbits similarly treated, when the galvanic power was transmitted along the nerve, no difficulty of breathing occurred, though the voltaic action was kept up for twenty-six hours. The rabbits were then killed, and the parsley found in a perfectly-digested state Hence, it appears that the galvanic energy is capable of supplying the nervous influence, so that, while under it the stomach, otherwise inactive, digests food as usual.

The same experiment was tried upon dogs, with like results—the battery-current, however, never being so strong as to occasion painful shocks.

In the case of extreme alcoholism, Faradism was applied as in the former case, but with this difference, that the poles were reversed, with a view of obtaining the sedative instead of the tonic effect of the current. After a few hours' application I was enabled to subdue the irritation, so that this, with the use of large and frequent doses of digitalis, produced quietude, sleep and healthier action of the emunctories.

I have no hesitancy in stating as my belief that, were these batteries generally recommended to be placed at life-saving stations, etc., many of the apparently drowned could be saved, even after all semblance of animation had ceased, or even as long as there remained one spark of vitality and heat in the great nerve-centers.

This paper would be incomplete if I failed to refer to the electro-motor properties of the animal body, in order that we may have a clearer understanding of the changes produced by the action of the electric current on the various animal tissues.

E. Du Bois-Reymond "was the first to succeed in demonstrating the presence of specific muscle and nerve currents, by the deflection of the magnetic needle;" and it was he, also, who ascertained that "if the nerve or muscle be excited by electric currents, or by mechanical or chemical irritants, so that the first is rendered physiologically active, and the latter caused to contract, and then placed at two symmetrical points in connection with the galvano-multiplier, a less deflection of the needle is produced than when the nerve or muscle is in a quiescent state. This is called the negative variation of the current." The conclusion arrived at by Du Bois-Reymond was that nerve and muscle contain innumerable positive and negative electric molecules, which move with great regularity throughout the tissue.

Then, perchance, the power of electricity, in these cases, may be to restore the suspended electrical forces of the body to a normal condition, and thus reanimate the failing vitality.

The toxic condition produced by chloroform may be remedied much in the same way; that is to say, by appealing to the vaso-motor centers through the pneumograstic axis.

For experiments illustrating the influence of anæsthetics on the vaso-motor centers, permit me to extract from a late and able paper by Drs. Bowditch and Minot, read before the Boston Society of Medical Science, and reported in the Boston Medical and Surgical Journal, of May, 1874:

"Anæsthetics, in producing insensibility to pain, accomplish such results by antagonizing the effects of irritation of sensitive nerves. One of the most constant physiological results of irritation of a sensitive nerve is a rise of arterial blood-tension, due to a reflex stimulation through the vaso-motor centers of the muscular walls of the smaller arteries; especially. those of the intestines. It is ascertained that, in the majority of cases, the rise of blood-tension, consequent upon the irritation of the saphenous nerve, is less marked when the animal is under the influence of ether than when the anæsthetic is not used.

"The first object was to determine the effects of anæsthesia on reflex-rise of blood-tension. This was accomplished in the following manner:

"An animal, being placed on the operating-table in the supine condition, a solution of curare was injected into the jugular vein, when paralysis ensued. When the respiratory movements ceased, the trachea was connected by means of a glass canula, inserted into it with the apparatus for artificial respiration, which was so adjusted as to imitate, as closely as possible, the normal respiratory rhythm. A canula was then placed in the carotid artery and connected with a mercury manometer, carrying a pen, by means of which the blood-tension was recorded on a long strip of paper which was kept in uniform motion by clock-work. The saphenous nerve was then placed upon electrodes.

"The irritation of the nerve was produced by closing the currents, by means of a key provided with a pen, thus recording the blood-tension, which could be seen at a glance. After the anæsthetic had been administered, the nerve was again irritated. The blood-tension was notably decreased, and so continued to be as long as these experiments were tried.

"But far more constant and obvious were the results obtained from chloroform. Here the irritation of the saphenous nerve caused a less marked rise in the blood-tension, and sometimes there was no rise whatever."

These facts present to my mind the clearest evidence in favor of the electrical remedy in cases of deep chloroform toxæmia, and the propriety of having accessibility to a Faradic instrument, complete and ready for immediate use, in chloroform administrations. It is also more than probable that electricity would be serviceable in many cases of still-birth.

A youth, aged seventeen years, while bathing, suddenly disappeared, and was beyond the aid of spectators or other bathers. "After awhile, the tide drifted him ashore far below the place he was last seen; he was taken up for dead, and a barrel made ready for the old process of resuscitation." In a short time, Dr Hastings, of Philadelphia, arrived with a battery and applied the electric poles "to his stomach and back, thus contracting the muscles of the stomach, causing the water to pour from his mouth; then inflated his lungs, and injected stimulants and medicine into his bowels. When he first examined the body there was no heart beat, no pulse, no circulation whatever. In less than an hour he had caused an artificial circulation through the heart, and he breathed; and, shortly after that, was screaming for help with all the agony of a drowning man. After six days, he has been restored to his right mind, but his memory of the event is gone, as much so as if his life had been entirely cut off. It is probable that, after awhile, as strength returns and old associations surround him, the memory of the fearful event will return."

Dr. Scheling reports, in the New York Medical Journal, that, after several hours' continued use of Dr. A. Murray's double-celled electrical apparatus, he succeeded in saving the life of a patient who had taken seven and a half grains of morphine.

Says Dadney:

"To obtain an efficient antidote for chloroform narcosis has long been a great desideratum. It is the general belief at the present day that ether is safer than chloroform; but, in many respects, the latter is to be preferred. It produces its effects much quicker than ether; its vapor is not inflammable as is that of ether, and it does not generally cause the feeling of suffocation which ether does."

Various means have been used to counteract the ill effects of chloroform when they supervene, depending on the character of the dangerous symptoms. Electricity, cold affusions, and artificial respiration have been the means chiefly employed for that purpose. All of these are possessed of considerable efficacy in certain cases.

We can pursue our studies to better advantage, however, by considering the modes in which chloroform kills. According to Gross (System of Surgery, vol. 1st, p. 563) it may cause death in four different ways, viz.:

1. By coma.
2. By asphyxia.
3. By syncope.
4. By gastric irritation.

1. Coma, as a mode of death from chloroform, occurs chiefly in old and debilitated individuals, or in those whose constitutions have been weakened by alcoholic or venereal excess. It is probably owing to the absorption of the chloroform and its powerful sedative influence over the brain and spinal cord. The action of the heart usually ceases with or before the cessation of respiration.

2. Death by asphyxia is usually caused by the want of a proper proportion of atmospheric air with the chloroform vapor. The air-cells become loaded with the vapor of the chloroform, and the blood is deprived of the normal quantity of oxygen. Only our surgeons of the present day, so far as we are informed, believe in thus cutting off the supply of air and overwhelming the patient with chloroform. The respiration usually ceases first, the heart continuing to beat some time longer.

3. Death by syncope is usually due to paralysis of the heart. The countenance, when death is threatened in this manner, is usually very pale and the pulse very weak and fluttering. The action of the heart ceases before the cessation of respiration.

4. Death from gastric irritation is very rare, and usually occurs gradually.

The second and fourth modes of death need not engage our attention further in this article. The first and third, which have some features in common, demand further consideration from us.

In both of these the heart's action ceases before the cessation of respiration; and in both some powerful stimulant is required.

Having thus examined briefly the modes in which chloroform may kill, and having seen that, in two of the modes at least, nervous stimulants are indicated, it is advisable that we should examine into the physiological properties of nitrite of amyl.

In 1864, Dr. B. W. Richardson reported to the British Association for the Advancement of Science that "the nitrite of amyl was, as an excitant of vascular action, the most powerful agent physiologically yet discovered." This report excited considerable interest in this salt, and its physiological properties were made the subject of study and experiment by several physiologists. The conclusions at which they arrived were entirely at variance with those of Richardson with regard to its action as an excitant of vascular action. Dr. H. C. Wood, of Philadelphia, undertook a series of experiments to determine which of the two accounts as to its action was correct; which experiments, like those of Gambee and Rutherford, tended to prove that it was a cardiac stimulant.

In the summer of 1872, a case occurred in the practice of my friend, Dr. R. W. Nelson, of Charlottesville, a prominent feature of which was the occurrence of repeated attacks of syncope, which was of very long duration, and from which the patient could with difficulty be aroused. At the suggestion of Dr. J. L. Cabell, of the University of Virginia, nitrite of amyl was given by inhalation in the dose of four or five drops. Her face instantly flushed up and she recovered her consciousness almost immediately; and, on one occasion, I am informed by Dr. Cabell, when she seemed on the very verge of dissolution.

It may be well to mention here that the nitrite of amyl has no effect on the coagulability of the blood, so far as known.

Its beneficial action does not seem to be confined to those cases in which there is great pallor. I recently attended a lady suffering from præ-vertebral abscess, who frequently had attacks of insensibility, during which the heart's action was very weak (sometimes imperceptible) but the face was either natural or somewhat flushed. At Dr. Cabell's suggestion, I administered amyl by inhalation in the dose of three drops, with the effect of bringing her to consciousness instantly.

Arguing from the conclusions of Richardson, as to the action of amyl, and also from its action in Dr. Nelson's case, reported above, I thought it would prove of great value in some cases of threatened death from chloroform, and determined to test its value.

My experiments, it is true, have been very few in number, and are not sufficient to lead to any definite conclusion as to its value; but you will see that, in three instances out of four, it produced marked effects.

Experiment 1.—Adult cat. The administration of chloroform was begun at seven minutes after two o'clock; at a quarter past two the cat seemed thoroughly narcotized; the pulse was regular and strong, and breathing normal; at nineteen minutes past two the heart's beat was very weak and irregular, and the breathing very slow and labored; at twenty-one minutes past two the heart's impulse was almost imperceptible, respiration gasping and at long intervals. Dropped five drops of amyl on a chip and held it to her nose; at twenty-three minutes past two the heart was beating stronger and more regularly; twenty-five minutes past two heart beating with great force, and breathing was hurried. She tried to move at this stage, but seemed to have lost all power over the voluntary muscles, except those which move the head from side to side. which responded slightly. At half past two she could move her fore-legs and body, but dragged her hind legs. At twenty minutes to three she had entirely recovered.

In this case the chloroform was given gradually, and mixed with a large proportion of atmospheric air.

Experiment 2.—Adult dog, (terrier). The administration of chloroform was begun at twenty-three minutes past seven o'clock, p. m. At half-past seven the dog was howling and scuffling, and passed his fæces. At twenty-two minutes to eight the narcosis was complete; the pulse and breathing were regular. At eighteen minutes to eight the pulse was very weak; at fifteen minutes to eight pulse almost imperceptible, breathing slow and labored. At fourteen minutes to eight injected five minims of amyl, hypodermically; twelve minutes to eight heart beating violently, breathing quick, animal still apparently unconscious; seven mintes to eight could move his head from side to side, but could move no other part of his body. He was then put out of doors; it was winter. At eight o'clock he seemed to have entirely recovered. The chloroform vapor was mixed with air and given slowly.

Experiment 3.—Adult dog, (terrier). Chloroform was given till his heart beat was almost imperceptible, and respiration at long intervals. Injected five minims of amyl; in two minutes his heart was beating with great force, and his breathing was very quick. There were present Drs. Nelson, of Charlottesville, Va., and Shelton, of Galveston, Texas. The chloroform was mixed with air and given slowly.

Experiment 4.—Half-grown puppy. The administration of chloroform was begun at a quarter past eleven, and all air was excluded; eighteen minutes past eleven dog was howling and trying to escape; twenty minutes past eleven poured on more chloroform; at twenty-three minutes past eleven pulse weak and breathing very faintly. Stopped the chloroform, and injected five minims of nitrite of amyl, and began artificial respiration, but could not arouse the animal in any way or get the heart to act. At half-past eleven I took out the heart, the right cavities of which were filled with venous blood, and dropped on four or five minims of amyl. This produced contraction.

In the first three experiments the chloroform vapor was mixed with air and given slowly, in the fourth all air was excluded and the animal overwhelmed with a large quantity of vapor at once.

In the first three experiments, also, the heart's action was much more affected than the respiration, in the fourth respiration had ceased entirely before the amyl was injected.

In the first three experiments the amyl produced a decided increase in the frequency and force of the heart's beats, in the fourth no effect was produced.

I am informed that the nitrite of amyl has been used in one instance for chloroform narcosis in the human subject with success, but I did not see the report of the case myself, and therefore know none of the particulars.

My own experiments on animals, as I said before, are too few in number to lead to any positive conclusions, but I have brought the subject before the Society with the hope that some one more skilled than myself in physiological experimentation, would test the value of amyl in chloroform narcosis, and give the result of his experience to the profession.

This is the resume of anæsthetics to date. The learned Dr. Turnbull, of Philadelphia, will discuss the subject as to its latest aspect, and afford you full information as to his own views and experiments.—*Transactions Maryland and District of Columbia Dental Association.*

